# Supplementary material for: Asynchronous magnetic resonance elastography: Shear wave speed reconstruction using noise correlation of incoherent waves
Source: Magn Reson Med. 2022 Oct 27;89(3):990–1001. doi: 10.1002/mrm.29502 (PMC9792433; doi:10.1002/mrm.29502)
Supplement: Supplementary file 1 — DATA S1 MATLAB code used to generate the numerical simulations of Figure 1 [file MRM-89-990-s002.zip › k-Wave/helpfiles/focusedBowlONeil.html]

focusedBowlONeil :: Functions (k-Wave)


# focusedBowlONeil

Compute O'Neil's solution for focused bowl transducer.

## Syntax

```
[p_axial, p_lateral] = focusedBowlONeil(radius, diameter, velocity, frequency, sound_speed, density, axial_position, lateral_position);
```

## Description

`focusedBowlONeil` calculates O'Neil's solution (O'Neil, H. Theory of focusing radiators. J. Acoust. Soc. Am., 21(5), 516-526, 1949) for the axial and lateral pressure amplitude generated by a focused bowl transducer when uniformly driven by a continuous wave sinusoid at a given frequency and normal surface velocity.

The solution is evaluated at the positions along the beam axis given by `axial_position` (where 0 corresponds to the transducer surface), and lateral positions through the geometric focus given by `lateral_position` (where 0 corresponds to the beam axis). To return only the axial or lateral pressure, set the either `axial_position` or `lateral_position` to `[]`.

Note, O'Neil's formulae are derived under the assumptions of the Rayleigh integral, which are valid when the transducer diameter is large compared to both the transducer height and the acoustic wavelength.

Example:

```
% define transducer parameters
radius      = 140e-3;   % [m]
diameter    = 120e-3;   % [m]
velocity    = 100e-3;   % [m/s]
frequency   = 1e6;      % [Hz]
sound_speed = 1500;     % [m/s]
density     = 1000;     % [kg/m^3]

% define position vectors
axial_position   = 0:1e-4:250e-3;       % [m]
lateral_position = -15e-3:1e-4:15e-3;   % [m]

% evaluate pressure
[p_axial, p_lateral] = focusedBowlONeil(radius, diameter, velocity, ...
    frequency, sound_speed, density, axial_position, lateral_position);

% plot
figure;
subplot(2, 1, 1);
plot(axial_position .* 1e3, p_axial .* 1e-6, 'k-');
xlabel('Axial Position [mm]');
ylabel('Pressure [MPa]');
subplot(2, 1, 2);
plot(lateral_position .* 1e3, p_lateral .* 1e-6, 'k-');
xlabel('Lateral Position [mm]');
ylabel('Pressure [MPa]');
```

## Inputs

|  |  |
| --- | --- |
| `radius` | transducer radius of curvature [m] |
| `diameter` | diameter of the circular transducer aperture [m] |
| `velocity` | normal surface velocity [m/s] |
| `frequency` | driving frequency [Hz] |
| `sound_speed` | speed of sound in the propagating medium [m/s] |
| `density` | density in the propagating medium [kg/m^3] |
| `axial_position` | vector of positions along the beam axis where the pressure amplitude is calculated [m] |
| `lateral_position` | vector of positions along the lateral direction where the pressure amplitude is calculated [m] |

## Outputs

|  |  |
| --- | --- |
| `p_axial` | pressure amplitude at the positions specified by `axial_position` [Pa] |
| `p_lateral` | pressure amplitude at the positions specified by `lateral_position` [Pa] |

## See Also

`mendousse`
